# Pregnancy Nephritis

**Published:** 1887-02

**Authors:** E. R. Armstrong

**Affiliations:** Holly, N. Y.


### *PREGNANCY NEPHRITIS.*

By Dr. E. R. ARMSTRONG, Holly, N. Y.

Medical science has been cultivated with such intelligence and laborious activity during the last three or four decades, and its fruits so carefully garnered in our vast storehouses of professional literature, there might seem to be nothing left for the common laborer but to utilize what has already been gathered together. As co-laborers our experiences may differ in the use of the material on hand, and by comparison each may receive benefit, and thereby become better equipped for professional work and especially for those emergencies which every one is liable to meet.

In obstetric pratice affairs may run smoothly for a long time, when suddenly and perhaps unexpectedly the physician is brought face to face with abnormal conditions that imperil the life of a human being, conditions that require, on the part of the practitioner, accurate knowledge, self-control and prompt action to rescue his patient from impending death.

Perhaps the most fearful and dangerous manifestation of diseased action, incident to the pregnant and parturient states, is eclampsia, as a sequence of nephritis. Fortunately for child-bearing women this affection is not very common; but since it gives a maternal mortality of about thirty per cent. and a fœtal mortality of fifty per cent. it must awaken in every philanthropic mind an earnest desire for a better mode of treatment than we now have. If so desirable a point be ever reached it must come by a careful and earnest investigation of pathological theories, and by clinical experience.

Various speculations have been advanced on the etiology of pregnancy nephritis. In 1840 Rayer called attention to the disease and described its principal symptoms. Three years later Lever published the results of observations in the London Hospitals, showing that nine out of ten parturient women who had convulsions were albuminous. Frerichs, in 1851, first attributed *eclampsia parturientium* to uraemic intoxication, as the sequence of nephritis, and from that time to this few forms of renal disease have been more written about, and yet its etiology is still unsettled. I will not here venture upon a discussion of the various theories that have been announced, but proceed to narrate two or three cases that have come under my observation during the last three or four years.

Case I.—Four years ago I was hastily summoned to see Mrs. H., a near neighbor, in her first confinement. Arriving at the bedside I found her in a severe convulsion and an irregular practitioner in attendance. He informed me that he had been with the patient twenty-four hours, during which time the pains had continued with the usual regularity, but had lacked force so that but little advancement had been made towards delivery. He also informed me that she had been very nervous and had complained of headache and distress in her stomach ; that she was suddenly and unexpectedly seized with a fit, and notwithstanding he had given repeated doses of lobelia to arrest them, they had continued for three hours with increasing energy and frequency. Being requested to take charge of the case, I at once administered, hypodermically, one fourth grain of morphia and one hundredth of atropia, following this with an enema containing thirty grains of chloral hydrate and forty grains of potassium bromide. A digital examination was now made, and finding the os retracted beyond the fœtal head, the forceps were applied and delivery effected in about twenty minutes. A few minutes later the placenta was expelled, the mother meantime being in profound coma with loud stertor. The patient being changed to the usual position in bed, was again seized with a severe convulsion. When it terminated, another hypodermic injection of morphia and atropia was administered, after which there were no more convulsions.

The patient remained in a comatose condition ten hours, and several hours more supervened before consciousness was fully established and the mental faculties had attained their normal condition. The bladder had been emptied with a catheter previous to delivery, of eight ounces of dark colored urine. An examination gave a specific gravity of 1035 and sixty per cent. of albumen. Retention of urine with vesical irritability lasted a week, requiring the use of the catheter every six hours. Under the use of salines and iron the general anasarca, as well as the albumen in the urine, rapidly disappeared, and in three weeks the patient was about the house. I learned from the family that for eight weeks previous to confinement the patient suffered from vertigo, headache, impaired vision, nausea, dysuria and œdema of the limbs and face. She was under the observation of her physician during this time, but no prophylaxis against eclampsia was used.

Case II.—Mrs. A., aged 27, primipara. I was called in consultation with Dr. B., last December, to see this lady, who had nearly completed her seventh month of gestation. Without any warning to her friends she had been suddenly prostrated with convulsions without any sign of labor. There had been several severe convulsions before my arrival, with coma during the intervals. Her condition plainly indicated pregnancy nephritis. There was œdema of the face and extremities with general anasarca. I learned that the patient had complained of vertigo, headache and nausea, and that the urine had been scanty and high colored. A sample was examined and found to contain seventy per cent. of albumen, with a specific gravity of 1036. A brisk purgative was administered *per orem* and thirty grains each of chloral hydrate and potassium bromide in two ounces of starch water as an enema. The latter was repeated in three hours, after which the paroxysms ceased and did not return. Saline laxatives, sufficient to keep the bowels open, with tincture of iron and bromide of potassium were administered during the remaining period of gestation, when labor came on, and she was delivered of a dead child without artificial aid. There was still œdema of the face and limbs with albuminous urine. The eliminative and tonic treatment was continued. At the end of twenty days no trace of albumen was found in the urine and the other abnormalities had likewise disappeared. She soon regained her usual standard of health, which has continued to the present time.

Case III.—Mrs. C., aged 22, primipara. In June last this lady had completed seven-and-a-half months of gestation, when I was called to see her. She was confined to the bed a portion of the time. There was cerebral disturbance in the form of headache, vertigo and impaired vision, with gastric irritability and constipation. The limbs, face and body were anasarcous to an unusual degree.

I obtained what urine had been voided in twenty-four hours and its analysis showed only 450 cubic centimeters in amount, dark with a specific gravity of 1035 and 30 per cent. of albumen. St. Vapor baths, on alternate days, were ordered, the bowels were kept open by a saline draught an hour before breakfast, and twenty minims of the tincture of the chloride of iron given after meals and at night. Under this treatment the cerebral and gastric disturbances were relieved, and the anasarca somewhat diminished. Six weeks more completed the period of gestation, when she passed through a natural labor, giving birth to a ten-pound, healthy boy.

In comparing these cases we observe that the symptoms of pregnancy nephritis were pronounced in each. In the first the morbid train of events culminated in eclampsia during labor at term ; in the second, six weeks before labor came on ; in the third case the convulsive explosion *only* was wanting. In all three the danger signals were present. In the last only, were these signals observed and therapeutic measures adopted with a view of arresting or modifying the morbid processes going on in the system. I have a strong suspicion that the convulsion explosion would have occurred in the last case had not measures been used to prevent it.

In pregnancy nephritis the primary lesion is undoubtedly in the kidney, and yet this organ does not seem to suffer any irreparable injury. At first its disturbance is mainly functional. Less urine is secreted, and what is secreted is of high specific gravity, high color and albuminous. As the case goes on there is generally cerebral and gastric disturbances, with a steady increase of dropsy and albuminuria until systemic toxæmia reaches the explosive point, when convulsions or premature parturition,or both together, ensue. If the patient survive the shock, not only the dropsy, but the urinary abnormalities likewise disappear with wonderful rapidity.

Pregnancy nephritis is perhaps more frequent than is generally supposed. It seems to me, judging from my own experience alone, that such disease is vastly more common than formerly, but there are so many sources of error in this that my observation is of no real value. The best statistics now show its presence in the ratio of one to twenty-three, appearing most frequently in primiparæ. But since these cases are not generally observed by physicians, until the very termination, the reports are quite unsatisfactory. The frequency, however, of nephritis, with its possible results, and the obscurity often of its general symptoms, should show how very important it is that the condition of the patient during pregnancy be carefully watched, especially in primiparæ. The victims of uræmic toxæmia are already far too numerous, since early detection would, in many instances, enable us to avoid a fatal issue. My obstetric record, during the last twenty years, contains ten cases of pregnancy nephritis that I have attended. Eight of these had eclampsia during parturition, one six weeks before, and the tenth presented all the precursory signs, which seemed to yield to treatment. One of the ten died, nine recovered. The case that proved fatal was in labor twelve hours, and had ten convulsions before my arrival.

With this limited clinical experience it would be presuming to attempt to formulate a course of treatment for others. If we turn to our authors for instruction on this subject we find it hard to reconcile the opposite views advanced by our most eminent men. Hodge, Gooch, Ramsbotham, Dewees, Meigs tell us to depend on the lancet. Meigs, on one page, says: "the only real resource in the puerperal convulsion is the use of the lancet, and nothing but the lancet is worth your confidence." On another page he tells us "the pregnancy ought to terminated in order to put a stop to the malady, therefore deliver as soon as possible." A wonderful contradiction in almost the same breath. On the other hand Trousseau, Leishman, Lusk and others declare "that indiscriminate bloodletting is a monstrous evil." Again, there are many practitioners of ability and experience who rely chiefly, if not entirely, upon chloroform as the remedy for this affections. There are still others who as strongly recommend morphia, chloral and the bromides. With such conflicting opinions and practice, I know of no better way than for each one to weigh the whole matter well and mark out his own course. Mine has already been indicated in the cases given. To be more explicit, I would; say, first ascertain the condition of the patient during the later part of pregnancy, when practicable, especially in primiparæ, and if there be signs of nephritis put the patient under treatment, with a view of avoiding convulsions. Second, if convulsions occur before the time of gestation expires control them with drugs if possible, otherwise induce premature labor. Third, if the attack be coincident with labor, and the patient strong and plethoric, bleed, purge and put the patient under the influence of chloral hydrate, giving fifteen to twenty grains every two hours until the convulsions cease. Fourth, if the patient be feeble and anæmic omit the bleeding. The bromides may be used with good effect, likewise morphia hypodermically. If the condition of the patient prevent the administration by the mouth use enemata. Fifth, as to the question of effecting speedy delivery. I am aware that it is a very common practice to expedite delivery by every means available, in many instances using severe and forcible measures to accomplish that object. In my humble opinion this practice is highly reprehensible, for if the *os* is not fully dilated and the child well down in the pelvis it will be immensely safer for the patient to keep her under the influence of chloral and morphia until the forceps can be easily applied, instead of forcibly dilating the *os* and turning the child, or dragging it away with instruments. I would not hesitate, however, in using the forceps as soon as it can be done with safety to both mother and child. We have remedies by which we can keep the patient easy and comfortable, and, therefore, we need be in no hurry to use violent or extreme measures.

